# Assessment of Dental Anxiety in Children: Reliability and Validity of the Questionnaire to Assess Dental Anxiety in Children (QADA-C)

**DOI:** 10.3390/dj12020023

**Published:** 2024-01-26

**Authors:** Jutta Margraf-Stiksrud, Klaus Pieper

**Affiliations:** 1 Department of Psychology, Philipps-University, 35032 Marburg, Germany; 2 Center for Dentistry, Oral and Maxillofacial Medicine, Philipps-University, 35039 Marburg, Germany

**Keywords:** children, dental anxiety, assessment, questionnaire, facet approach

## Abstract

This study presents a measure to assess dental anxiety in children. To gain a better understanding of children’s fear and anxiety in the dental setting, instruments to gather data with high quality are necessary and missing, especially in the German language. Based upon the facet approach, the Questionnaire to Assess Dental Anxiety in Children (QADA-C) includes items concerning anxiety reactions in thinking, behavior, and feelings in the form of short statements. The item formulation was adapted for children of the target age (9–11 years), and items were presented with pictures of dental situations. Item and reliability analyses in a sample of 1019 children showed the good quality of the instrument (Cronbach’s alpha = 0.89), and the validity indicators revealed its ability to differentiate high-anxiety, low-anxiety, and non-anxious children with an overall sum score. This score was proven to correlate with oral health criteria (decayed/missing/filled teeth, oral health behavior, oral health knowledge). Its easy administration and appropriateness for children make the questionnaire applicable in the dental office for clinical purposes as well as in research contexts.

## 1. Introduction

Anxiety and fear reactions in the dental setting are common and vary in intensity. Phobic forms occur in about 5% of subjects in most of the investigated populations, who risk the avoidance of dental treatment in spite of oral health problems [1,2]. Less-intense feelings of anxiety are more frequent and reported as causing strain by nearly 50% of all patients when visiting the dentist. Anxiety and fear in patients are a challenge for dentists and a risk to trouble-free treatment [3]. To understand the origin and development of dental anxiety, several factors have proven to be relevant. Melamed, a pioneer in investigating this phenomenon, wrote in 1979, summarizing: “Many researchers … have uncovered combinations of family experience, attitudes, traumatic facial experience, low pain tolerance, and high anxiety which underlie dental fear” ([4], p. 172]). Today, a cognitive–behavioral model of dental anxiety is widely accepted where negative expectations, cognitive appraisal processes, and a lack of adequate coping behavior support feelings of worry and anxiety [5,6]. 

Because experiences with dental treatment as well as the learning of coping behavior start early in childhood, adult patients often report traumatic events with the dentist in childhood as a cause of their fear [7]. Actually, patients with the onset of dental anxiety in (young) adulthood are much less frequent, and personality factors (vulnerability, general anxiety) seem to be more important than conditioning processes in those cases [8,9]. Therefore, parents, dentists, teachers, and staff persons should provide realistic information about dental procedures, demonstrate dental instruments, and help relax and distract the children when they experience unpleasant sensations [10].

When dealing with children who are already anxious, the dentist (or other professionals) should be capable of evaluating the child’s condition and assessing the intensity and source of his/her anxiety to decide upon an effective management strategy, which enables the necessary dental treatment. The first steps in this regard are careful observation of the child’s behavior and physical state, accompanied by sensitive questions about his/her feelings and past experiences and interviewing parents as an additional source of information. This approach corresponds to established diagnostic principles in that it uses several methods to enhance the quality of information and avoid misjudgments. Moreover, correlations between self-reported anxiety, behavioral and physiological signs of fear, and parental information are often low [11,12,13,14] and afford additional investigations to understand and evaluate the meaning of these discrepancies. Therefore, in several reviews, self-report questionnaires are emphasized as a gold standard for assessing children’s dental anxiety [11,15,16]—not only in children but also for adults [3,17].

There is a tradition of self-report methods for children that are commonly used and analyzed, namely, the Children Fear Survey Schedule (Dental Scale, CFSS-DS, [18]), the Children’s version of the Dental Anxiety Scale (MCDAS, [19]), and the Dental Fear Survey—Children Scale (DFS, [20]). All scales show satisfying overall reliability but are criticized for a lack of theoretical foundation, transferring from adult questionnaires more than creating children-specific constructions, and limited applicability based on the item contents, which favor only qualified aspects of dental anxiety [11]. Therefore, the development of a new measure for investigating anxiety and fear in children for research purposes as well as practical use in Germany, the Questionnaire to Assess Dental Anxiety in Children (QADA-C) (Fragebogen zur Erfassung von Zahnbehandlungsangst bei Kindern, FEZ-Ki), was preferred over an adaptation of the aforementioned established instruments.

A theoretical framework, a children-specific item formulation and design of the questionnaire, and easy and economic administration were the main principles of the developmental process [21]. The QADA-C showed satisfying practicability, reliability, and validity in previous studies with 8- to 13-year-old children (summarized in [21]). However, the sample sizes were small (each under 100 children), and an examination of construct validity (factor structure, replication of findings concerning dental anxiety in children) is pending. 

The aims of the current study are to replicate the reliability results of the QADA-C and investigate its construct validity in an extended sample of 9- to 11-year-old children. These objectives were pursued as a part of a larger research project. 

## 2. Materials and Methods

Further validation of the QADA-C took place in the context of a study to evaluate an intensified preventive program for children with increased caries risk that is supported by the German Federal Government (Grant No. BMBF 01EL0617) [22,23]. This study was approved by the Committee for Ethics of the medical faculty, University of Marburg, Germany (file number 200/06). One of the outcome variables used to test the effectiveness of the prevention program was dental anxiety, along with oral-health-related knowledge, oral hygiene behavior, and attitudes of the investigated children and their parents, as well as dental parameters like health status (DMF-T, plaque indices) and prevention behavior (using fluorides, visiting the dentist regularly, participating in prevention measures). The study design included a control group, who did not receive intensified but rather basic preventive care. A total of 2995 children participated in this study in the years 2007–2009 (n = 768 study group, n = 2227 control group). Of these, 1051 were first graders, 1019 were fourth graders, and 925 were sixth graders, with a mean age of 6.7 years (SD 0.83), 9.7 years (SD 0.77), and 12.1 years (SD 0.65), respectively. Regarding gender, 51% of the children were girls (n = 1530). The participation rate was 69.9%, and no data about the non-consenting families were available. The main results of this study were presented in several publications (e.g., [22,23,24,25]).

### 2.1. Procedure

The QADA-C was administered as part of a questionnaire booklet (supplementary material) in the school setting. All children and their parents were previously informed about the study aims and declared consent. Two investigators, a psychologist and a dentist, distributed the booklet to the children and explained and supervised answering. Afterward, the dentist performed an oral examination of each child in a separate room, prepared with a mobile dental examination unit, while the psychologist assisted and observed the child’s behavior. 

### 2.2. Participants in the QADA-C Validation Study

As the aim of the current study is the applicability of the QADA-C in 9–11-year-old children, only the data on the 1019 fourth graders were used here.

### 2.3. Measures

#### 2.3.1. Dental Anxiety

**Figure 1:**
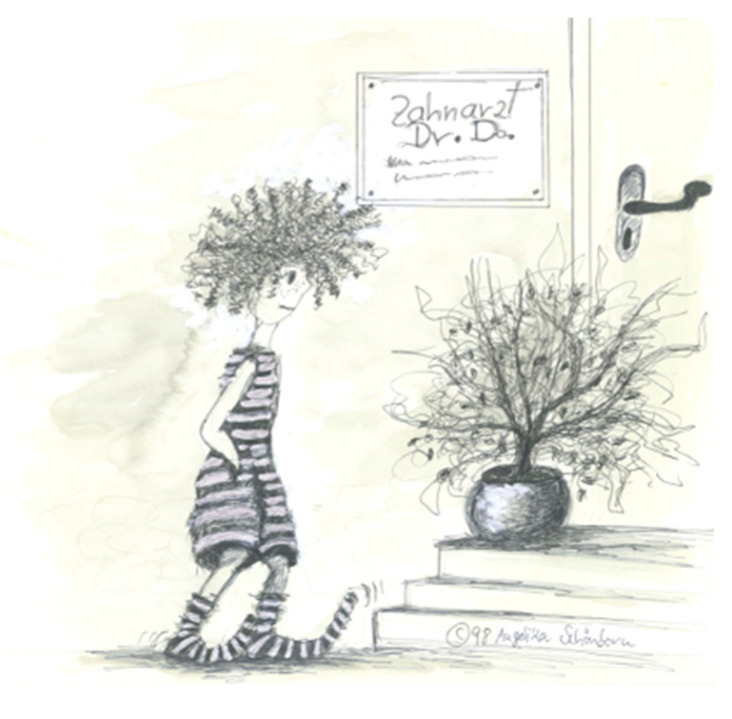
Example for the pictures of QADA-C: situation 2, “On the way to the dentist”.

As a theoretical framework, the QADA-C is based on a facet approach according to Stouthard, Mellenbergh, and Hoogstraten [26]. Anxiety reactions are diverse and involve feelings and thoughts, physiological alterations, and overt behavior (reaction facet). They are triggered by dental stimuli and stressors (situation facet) and intensify while approaching these situational cues (time facet). Following these theoretical presumptions, the collection of items concerning anxiety reactions was associated with five situations representing scenes of a visit to the dentist (evening before an appointment, on the way to the dentist, in the waiting room, sitting in the dental chair, and treatment with instruments; see Figure 1 for an example). All items were selected according to expert ratings about suitability in representing an anxiety reaction and appropriateness for the age group and tested by children regarding comprehensibility and clarity. Item analysis resulted in 5 items per situation (25 items in total). In each situation, different anxiety reactions were presented. Children either agreed or disagreed with the statements. 

Administration time of about 10 min and pictures as visualization of the time and situation facet enhance the compliance and motivation of children. Internal consistency revealed a mean Cronbach’s alpha coefficient of 0.83, and correlations with measures of general anxiety (Children Fear Survey Schedule—CFSS, and KAT (“Kinder-Angst-Test”, Children Anxiety Test, [27]) proved concurrent validity (CFSS: r = 0.48; KAT: r = 0.34, not corrected for attenuation). Along with the QADA-C (for items see Table 1), the children rated their anxiety in each of the five situations using a rating scale (endpoints: “no anxiety” and “very much anxiety”) with 10 units (not verbally specified).

#### 2.3.2. Measures to Prove Validity: Dental Health Questionnaire and Dental Health Status

The examination booklet contained a 10-item questionnaire about dental health knowledge (multiple choice questions) and 54 items concerning oral hygiene behavior and attitudes (for further information concerning these questionnaires, see [23,24]). Decayed (D), missing (M), and filled (F) teeth (T) (due to caries) were registered during the dental examination using the International Caries Detection and Assessment System (ICDAS II) [28,29]. The dentist was specially trained for this purpose. As the most relevant outcome variable, D_3–6_MF-T was used, meaning a calculation of the “D”-component via ICDAS scores 3–6 [24]. All results concerning the distribution and other descriptive statistics of these measures are published elsewhere [22,23,24,25].

### 2.4. Data Analyses

All analyses were performed with SPSS Version 17.0 (IBM, Chicago, IL, USA). Item analysis included item difficulties, item–test correlations with part–whole correction, and reliability analysis (Cronbach’s alpha). An exploratory factor analysis was conducted (principal factor) to show structural properties of the newly developed instrument as a basis for construct validity. For mean comparisons of the QADA-C scores in different groups (gender, situations, membership to the study, and control group in the main study), parametric methods were used to exploit the information in the data on this cross-sectional design (*t*-tests and analyses of variance). Considering the sample size (central limit theorem), this seemed to be appropriate. The significance level was set at alpha = 0.05.

## 3. Results

### 3.1. Descriptive Data, Item Analyses, and Reliability

**Figure 2:**
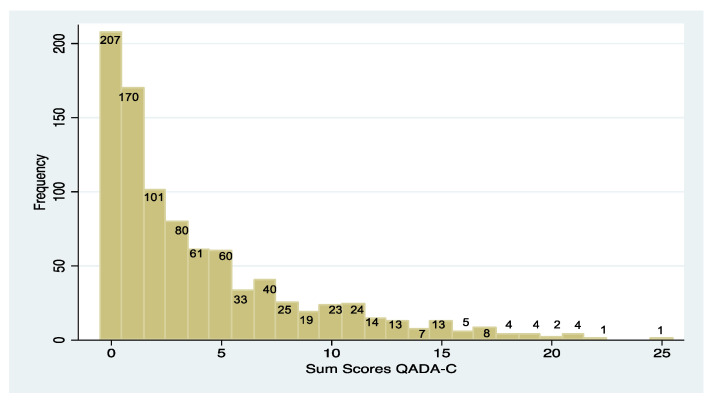
Mean distribution of the QADA-C sum scores (Q_1_ = 0, Md = 2, Q_3_ = 6, upper 10% > 11, upper 5% > 13).

The analyses were based on n = 919 completely answered questionnaires. The mean of the QADA-C sum score was 3.98 (SD 4.57) with a median score of 2. Figure 2 shows the distribution of means. Of the children, 67.4% attained a score equal to or below the group mean, while about 5% of the children ranked over two standard deviations above the group mean (score > 13), leading to a skewness of 1544. 

The item analysis showed corrected item–total correlations (Table 1) ranging between r = 0.30 (item 5) and r = 0.62 (item 11). Item 17 (“I want someone of my family to stay with me”) has the lowest item difficulty (53.3); Item 16 (“I must cry when I see the dentist”) has the highest (2.4). Overall, the internal consistency was proven to be high (Cronbach’s alpha = 0.89). 

**Table 1:** QADA-C items, item–test reliability, and factor loadings.

Situations		Items	Mean	Standard Deviation	Item–Test Correlation ^1^	Factor Loading
Situation 1 “Evening before an appointment”	1	I cannot fall asleep while thinking about my dental appointment	0.79	0.41	0.555	0.591
	2	I have a tummy ache	0.92	0.26	0.393	0.425
	3	I wish I must never go to the dentist again	0.87	0.33	0.393	0.456
	4	I am so excited that I cannot fall asleep	0.78	0.41	0.533	0.552
	5	I cry because I have to go to the dentist	0.97	0.16	0.302	0.351
Situation 2 “On the way to the dentist”	6	I get more and more excited when I approach the dental office	0.70	0.46	0.552	0.564
	7	I would like to run away	0.90	0.30	0.552	0.623
	8	I believe something terrible will happen	0.89	0.31	0.519	0.567
	9	My knees are trembling	0.89	0.31	0.566	0.593
	10	I have a very dry mouth	0.84	0.37	0.449	0.483
Situation 3 “In the waiting room”	11	I want to go home again	0.85	0.35	0.617	0.693
	12	I must always go to the bathroom	0.95	0.23	0.365	0.395
	13	I am getting sick	0.96	0.20	0.402	0.448
	14	I am getting nervous when the dental assistant calls on me	0.71	0.45	0.606	0.610
	15	I think it will get quite dreadful	0.91	0.29	0.565	0.629
Situation 4 “Sitting in the dental chair”	16	I must cry when I see the dentist	0.98	0.15	0.331	0.377
	17	I want someone of my family to stay with me	0.47	0.50	0.370	0.360
	18	I whish I would be far away	0.90	0.30	0.602	0.681
	19	I start to sweat	0.96	0.21	0.376	0.416
	20	When the dentist speaks with me, I start to stutter	0.94	0.23	0.473	0.528
Situation 5 “Treatment with instruments”	21	I cannot stand the noise of drilling	0.70	0.46	0.415	0.424
	22	I must think about what might happen	0.74	0.44	0.542	0.565
	23	I close my eyes tightly	0.74	0.44	0.368	0.371
	24	My heart beats very strongly	0.81	0.39	0.577	0.590
	25	I cling to the chair firmly	0.82	0.39	0.518	0.514

^1^ Part–whole corrected.

### 3.2. Construct Validity

#### 3.2.1. Situation Mean Distribution and Exploratory Factor Analysis

The mean scores (standard deviations (SDs) in parentheses) for the five situations were M_1_ = 0.65 (SD = 0.033), M_2_ = 0.79 (SD = 0.037), M_3_ = 0.63 (SD = 0.033), M_4_ = 0.76 (SD = 0.027), and M_5_ = 1.18 (SD = 0.044), representing the total amount of agreement with the five items of each situation. The last situation (during the dental treatment) provoked the most feelings of tension and anxiety. According to the MANOVA for repeated measurements (F [4,915] = 72.03, *p* = 0.001), the differences between the last and the four other situations were significant. Post-estimation contrasts further revealed significant differences between the two lowest means (the first and third situations) and the remaining two (the second and fourth situations). The scores of the anxiety rating scale, which represented an overall estimation of anxiety levels for each situation, increased from the first to the last situation (means: 1.89, 1.95, 2.00, 2.09, 2.31).

Concerning Kaiser–Meyer–Olkin (KMO) criteria (overall 0.92), the data fit very well for an exploratory (principal) factor analysis. Two factors showed an eigenvalue greater than 1 (6.82, 1.15). Factor loading patterns revealed no clear interpretation basis for two factors, even after (oblique) rotation. Therefore, the one-factor solution was accepted (see Table 1 for factor loadings).

#### 3.2.2. Gender Differences

Boys reported significantly less anxiety than girls, measured with the QADA-C (boys: M = 3.61, SD = 4.46; girls: M = 4.33, SD = 4.64; t = −2.38, *p* = 0.017, Cohen’s d = −0.15), which was also true for the anxiety rating scale (boys: M = 9.96, SD = 8.73; girls: M = 12.08, SD = 9.40, t = −3.599, *p* = 0.0003, Cohen’s d = −0.23).

### 3.3. Criterion Validity

Table 2 shows the intercorrelations between the QADA-C and the other measured variables. They indicate discriminant (knowledge, behavior) and convergent validity (anxiety rating). As expected, the correlation with anxiety rating is high (r = 0.723), while the correlations between the QADA-C results and dental knowledge or dental hygiene behavior are very small but significant (r = −0.122), and that between the QADA-C and dental hygiene behavior is not significant (r = 0.051). 

Table 2 also shows the correlations between the main indicator of oral health, the DMF-T-Index, and the QADA-C results. Oral health is slightly and similarly correlated to both anxiety measures (r = 0.139 and r = 0.133, respectively) but not to knowledge or dental hygiene behavior. The correlation pattern shows similar results for both the QADA-C and the anxiety rating, emphasizing the comparable meaning of the two measures. The correlations correspond in height and direction to expectations, as good health behavior and higher knowledge should be associated with lower anxiety. 

The QADA-C results were M = 3.48 (SD = 4.52) for the study group and M = 4.47 (SD = 4.61) for the control group, and the difference was significant (F = 4.65, *p* = 0.017, Cohen’s f = 0.17). Additionally, the anxiety rating showed the same pattern (study group: M = 9.77, SD = 7.95; control group M = 12.03, SD = 9.78, F = 5.101, *p* = 0.013, Cohen’s f = 0.18). 

## 4. Discussion

The reliability review and validity estimation of the QADA-C for fourth graders show overall satisfying results. The item–total correlations and the alpha coefficient correspond to former results in other, smaller samples and prove the rather consistent meaning of the 25 items. This matches with the findings of exploratory factor analysis, revealing a one-factor solution as fitting best to explain the variance in the answers. 

The descriptive data of the QADA-C show a common pattern of score distribution for dental anxiety ratings, though original scores are not often reported. Ten Berge, Veerkamp, Hoogstraten, and Prins [30] indicated that 14% of their sample of 2144 Dutch children was in borderline or clinical ranges (one standard deviation above the mean), while 66% scored below the group mean, as assessed with CFSS-DS. These data match the present data on the QADA-C and confirm a right-skewed distribution of anxiety levels in random samples.

An important indicator of the validity of the QADA-C is the high correlation with the anxiety rating, which verbalizes and quantifies the construct directly. As a usual procedure to discriminate traits from other relevant variables in a context, dental health knowledge and self-reported dental behavior and attitudes were used to prove discriminant validity. 

The anxiety reported in the QADA-C as well as the anxiety rating did correlate with oral health knowledge in fourth graders to a very small but significant extent, which might show the interrelation between emotional and cognitive reactions to this special (dental health) topic. Oral health behavior shows a small relationship only to the anxiety rating. At the age of 9–11 years, one could argue, oral hygiene behavior as well as visiting the dentist depends more on parents’ influence than on the autonomous decisions of the child. Therefore, good parental care might serve as a buffer for appropriate hygiene behavior even when a child tends to avoid dental activities because of anxiety, and vice versa. This can be an explanation for the very small coefficients. Interestingly, in spite of the described educational situation, the correlation between dental health (DMF-T index) and anxiety is more pronounced. This can be interpreted as strong proof of the validity of the QADA-C, because a correlation between dental health and anxiety is often verified and widely accepted. 

With the QADA-C, we were able to replicate the often-reported gender difference in anxiety in 9–10-year-old children in the anxiety rating results [31,32]. Girls tend to describe themselves as more anxious than boys. The proportion of highly anxious children, about 5% in our sample, also matches data from other studies (e.g., [2]). 

Because the QADA-C proved to be a reliable and valid measure for anxiety in our study, the benefit of using this questionnaire compared to other ones must be further discussed. Al-Namankany and co-authors [16] list a number of quality criteria for self-report scales in their review of widely used questionnaires (and observation scales). Besides classical criteria like reliability, validity, and objectivity, the authors underline the importance of easy and economic administration, data analysis, and interpretation for application in the dental office/clinic as well as for research purposes. They add the necessity of child-appropriate design concerning item formulation and layout to gain motivation and considering the cognitive development of the targeted children. Porritt and co-authors [11] use the term “developmental validity” to indicate an item selection that is adapted in language and terminology to the child’s comprehension of questions and response alternatives. Desirable contents should be a quantitative grading of anxiety, a ranking of anxiety-provoking stimuli, and information about beliefs and thinking patterns associated with feeling anxious and fearful [14]. In both reviews, none of the analyzed self-reports meets all these criteria. About nine self-report scales are discussed (somewhat different ones in either review), and their differential usefulness for several purposes and populations is considered. 

Of the above-mentioned criteria, the QADA-C meets most: it proved to be a questionnaire with a high acceptance by the children because of its short and age-appropriate items presented along with pictures visualizing the feared situations. As the five situations that are shown in the pictures represent a gradual approach to dental treatment, the mean score of each situation should increase, which was verified for the item scores as well as for the anxiety rating. The QADA-C’s concept does not enable the ranking of anxiety-provoking stimuli, for the situations represent several potentially anxiety-provoking stimuli at once. As a benefit of this approach, the user can further question the child individually about the situations she/he ranked as highly anxiety-inducing; therefore, the QADA-C might serve as a screening instrument to decide on additional diagnostic steps (anxiety stimuli hierarchy, parent interview, systematic observation). In this respect, the QADA-C delivers more valuable information than one-item questionnaires (e.g., anxiety rating) without overstraining the children. 

As the distribution pattern of the sum scores in this sample shows, the results of the QADA-C can also be used to obtain reliable information about the anxiety level of a child facing dental treatment. Scores above 2 indicate that the child is more anxious than 50% of his/her age group. Only 5% of the children reached scores above 13, so those results may apply to a highly anxious child. In professional pediatric dental care, the approach toward the child should take the extent of his or her anxiety into account [33]. Therefore, the assessment of the anxiety level with the QADA-C can uncomplicatedly lead to the selection of a strategy that is appropriate for the child: scores up to 2 do not require special measures, scores from 2 to 10 should lead to a more in-depth questioning of the child, and scores above 10 can be expected to disallow treatment unless special help is provided for the child.

### Limitations

The QADA-C is not able to discriminate thinking patterns, the physiological aspects of anxiety and fear, or overt behavior reactions. The data analysis did not show separate factors concerning the reaction facet. This corresponds to the results of Stouthard, Mellenbergh, and Hoogstraten [26] concerning the facet structure of their Dental Anxiety Inventory. Even in different samples of adults, specifically the reaction facet could not be found in confirmatory or exploratory factor analysis, while the time and situation facets were verified. A three-factor solution fitted best, representing a general factor of anxiety together with two (not independent) factors named “fear for dentist’s comments” and “fear of drilling, extraction and anesthesia”. Besides the formal differences between the DAI and the QADA-C (number of items, response scale, using pictures as situational cues), the cognitive differentiation processes of children who are about 10 years old have to be taken into account. Therefore, the general anxiety factor found in the QADA-C seems to be adequate for this age group.

As the QADA-C claims to assess dental anxiety as a trait, data about retest reliability are necessary to prove this capacity. Data from very small samples (under 20 children) show promising results (r_tt_ = 0.88 after two years) but need to be further complemented. Additionally, for future research as well as for practical purposes, QADA-C data should be gathered in an everyday clinical situation together with behavior reactions during dental treatment to show predictive validity. 

## 5. Conclusions

When used in an evaluation study with a large sample of children, the QADA-C proved to be a reliable and valid instrument. In 9–11-year-old children, it is easily administered in this group and also well accepted. The questioning supported by pictures enhanced the motivation of the children to answer the 25 short statements appropriately. The outcome of the measure is important information for the dentist to align the treatment adequately to the extent of the child’s anxiety. The QADA-C is suitable for assessing dental anxiety in children and in research contexts.

## Figures and Tables

**Table 2:** Intercorrelations of QADA-C and several validity measures (n = 821).

	Anxiety Rating	Oral Health Knowledge	Oral Health Behavior ^1^	DMF-T
QADA-C	0.723 **	−0.122 **	0.051	0.139 **
Anxiety Rating		−0.063 *	0.101 *	0.133 **
Oral Health Knowledge			−0.189 **	−0.055
Oral Health Behavior				0.059

^1^ Lower scores mean better oral health behavior; * *p* < 0.05; ** *p* < 0.01.

## Data Availability

The raw data supporting the conclusions of this article will be made available by the authors on request.

## Supplementary Materials

The following are available online at https://www.mdpi.com/article/10.3390/dj12020023/s1, A visit to the dentist.
