# Supplementary material for: Assessment of Dental Anxiety in Children: Reliability and Validity of the Questionnaire to Assess Dental Anxiety in Children (QADA-C)
Source: Dent J (Basel). 2024 Jan 26;12(2):23. doi: 10.3390/dj12020023 (PMC10887742; doi:10.3390/dj12020023)
Supplement: Supplementary file 1 [file dentistry-12-00023-s001.zip › dentistry-2647510-supplementary.pdf]

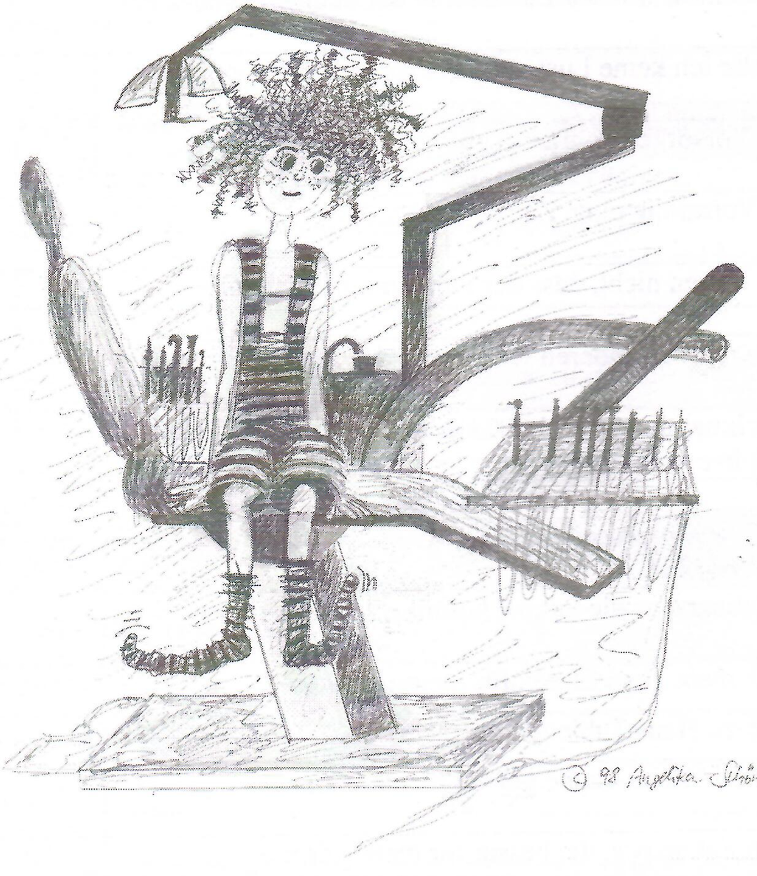

Ein Besuch beim Zahnarzt

© Jutta Margraf-Stiksrud

Auf den nächsten Seiten findest Du einige Sätze. Es sind Aussagen darüber, wie Du Dich vielleicht fühlst, wenn Du zum Zahnarzt gehst. Versuche bitte, diese Sätze der Reihe nach zu beantworten, ob sie für Dich zutreffen oder nicht.

Bitte beantworte **alle** Sätze und ohne lange darüber nachzudenken. Es gibt keine richtigen oder falschen Antworten. Wenn eine Aussage für Dich zutrifft, machst Du ein Kreuz in das Kästchen unter dem Wort „Stimmt".

Wenn sie nicht zutrifft, machst Du ein Kreuz unter den Wörtern „Stimmt nicht".

Beispiel:

	stimmt	stimmt nicht
Ich gehe gerne zum Zahnarzt.	☐	☐
Wenn Du also gern zum Zahnarzt gehst, kreuzt Du das Kästchen unter „stimmt" an.	☒	☐
Wenn Du nicht gern zum Zahnarzt gehst, kreuzt Du das Kästchen „Stimmt nicht" an	☐	☒

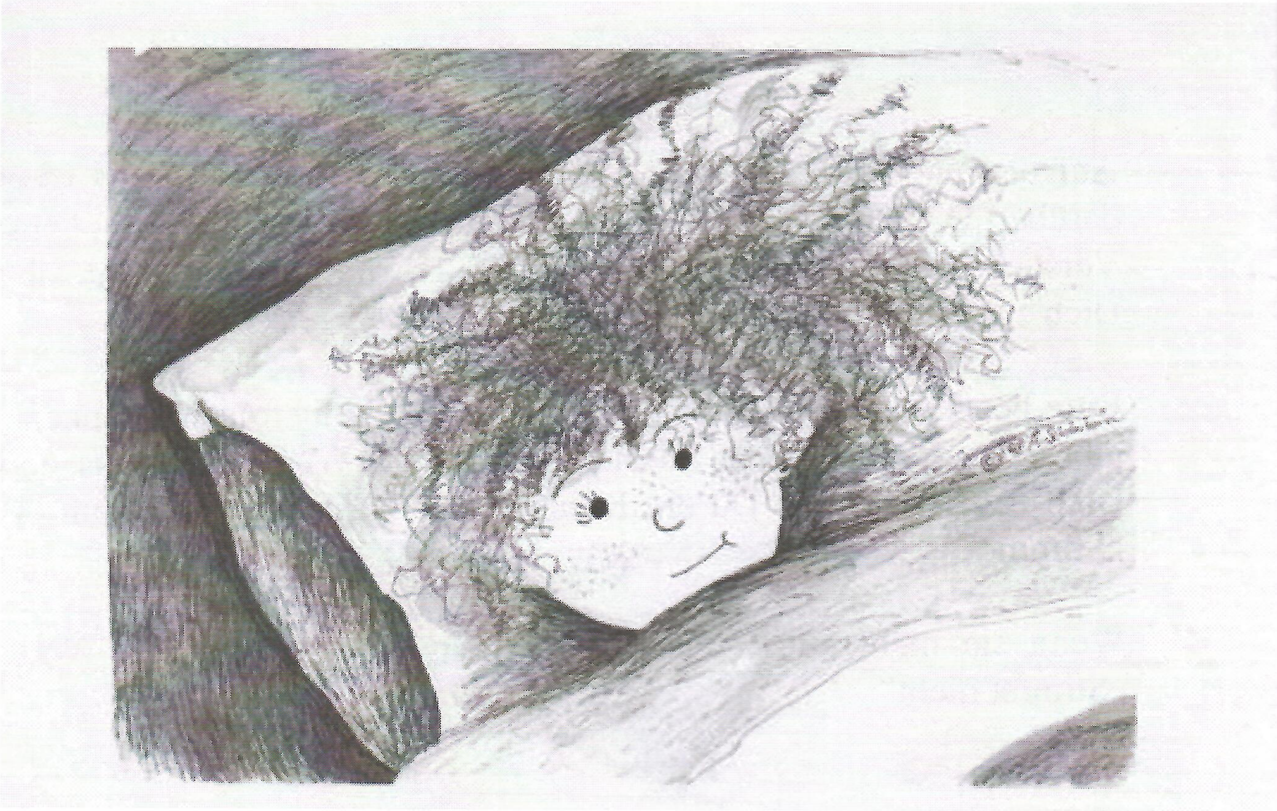

Stelle Dir vor, Du bist zu Hause und liegst im Bett. Im Zimmer ist es schon dunkel. Da fällt Dir ein, dass Du morgen zum Zahnarzt gehst. Kreuze an, was Du ***jetzt*** *denkst und fühlst.*

	stimmt	stimmt nicht
1. Ich kann lange nicht einschlafen, weil ich mir so viele Gedanken machen muss.	☐	☐
2. Ich habe Bauchschmerzen.	☐	☐
3. Ich wünschte, dass ich nie mehr zum Zahnarzt gehen muss.	☐	☐
4. Ich schlafe vor Aufregung schlecht ein.	☐	☐
5. Ich weine, weil ich zum Zahnarzt muss.	☐	☐

Bitte kreuze den Strich auf der Linie an, der zeigt, wie viel Angst Du in dieser Situation hast:

keine Angst ┼─┼─┼─┼─┼─┼─┼─┼─┼─┼ sehr viel Angst

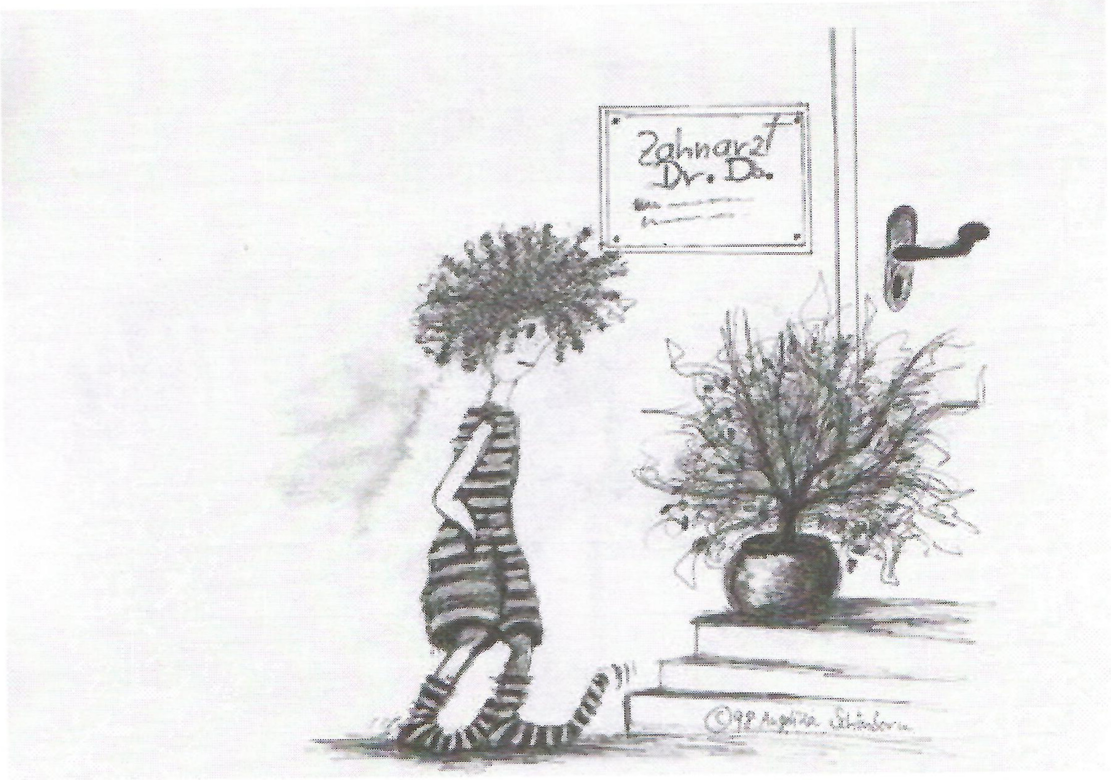

Stelle Dir vor, Du bist auf dem Weg zum Zahnarzt. Du kannst das Haus, in dem der Zahnarzt seine Praxis hat, schon sehen. Gleich wirst Du durch die Eingangstür gehen.
*Kreuze an, was Du **jetzt** denkst und fühlst.*

		stimmt	stimmt nicht
6.	Ich werde immer aufgeregter, je näher ich der Zahnarztpraxis komme.	☐	☐
7.	Ich hätte Lust wegzulaufen.	☐	☐
8.	Ich glaube, dass etwas ganz Schlimmes passieren wird.	☐	☐
9.	Meine Knie zittern.	☐	☐
10.	Ich habe einen ganz trockenen Mund.	☐	☐

Bitte kreuze den Strich auf der Linie an, der zeigt, wie viel Angst Du in dieser Situation hast:

keine Angst ├─┼─┼─┼─┼─┼─┼─┼─┼─┤ sehr viel Angst

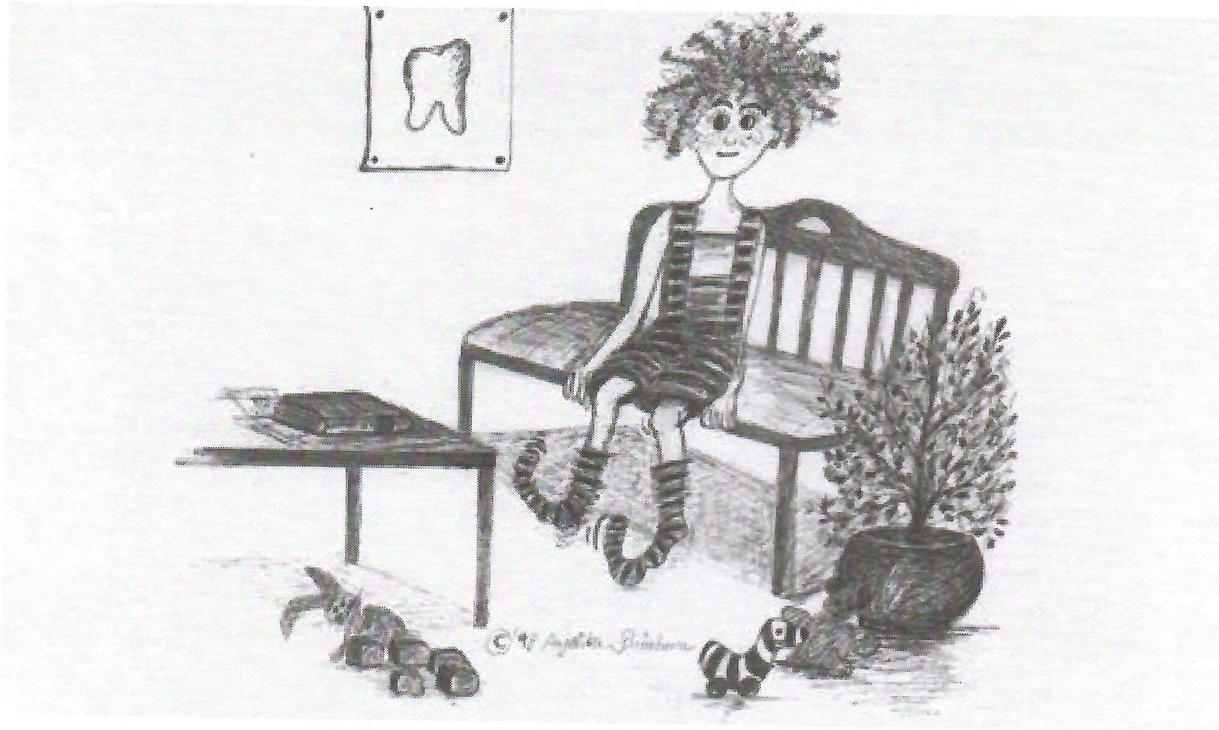

Stelle Dir vor, Du sitzt jetzt im Wartezimmer. Du bist noch nicht an der Reihe. In Deiner Nähe liegen Bücher, Zeitschriften und Spielzeug. Jeden Moment wird Dich die Zahnarzthelferin hereinrufen.
*Kreuze an, was Du **jetzt** denkst und fühlst.*

	stimmt	stimmt nicht
11. Ich möchte wieder nach Hause gehen.	☐	☐
12. Ich muss immer wieder zur Toilette.	☐	☐
13. Mir wird übel.	☐	☐
14. Ich werde nervös, wenn die Zahnarzthelferin mich aufruft.	☐	☐
15. Ich denke, dass es ganz furchtbar wird.	☐	☐

Bitte kreuze den Strich auf der Linie an, der zeigt, wie viel Angst Du in dieser Situation hast:

keine Angst |—|—|—|—|—|—|—|—|—| sehr viel Angst

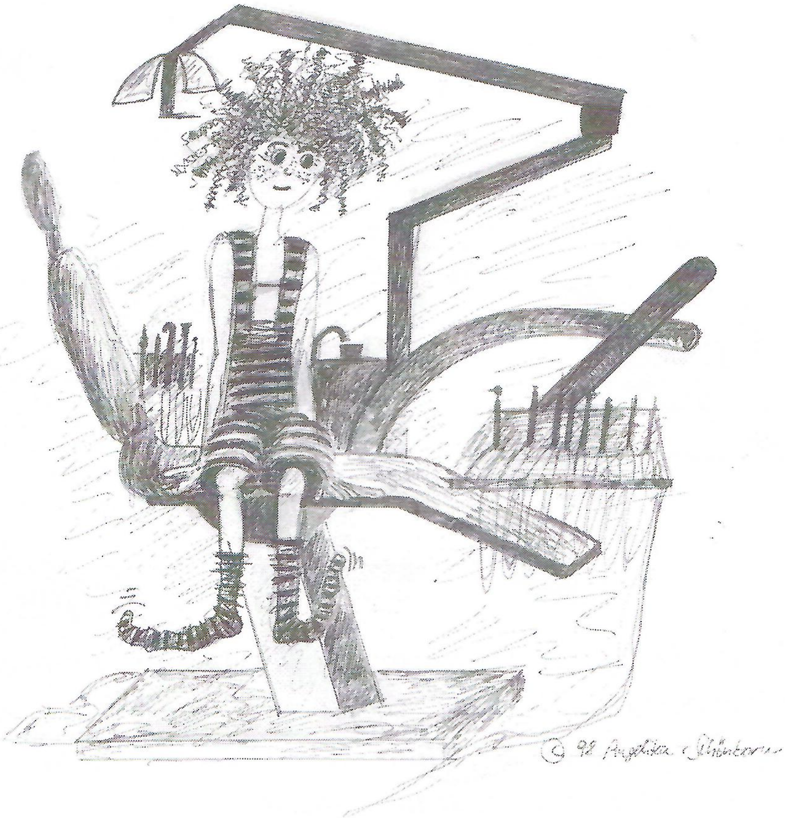

Stelle Dir vor, dass Du im Behandlungsstuhl sitzt. Es riecht nach Zahnarzt. Der Zahnarzt grüsst Dich. Du siehst die verschiedenen Instrumente. Gleich wird eins davon benutzt.
*Kreuze an, was Du **jetzt** denkst und fühlst.*

	stimmt	stimmt nicht
16. Wenn ich den Zahnarzt sehe, muss ich weinen.	☐	☐
17. Ich will, dass jemand von meiner Familie bei mir bleiben darf.	☐	☐
18. Ich wünschte, ich wäre weit weg.	☐	☐
19. Mir bricht der Schweiß aus.	☐	☐
20. Ich fange an zu stottern, wenn der Zahnarzt mit mir spricht.	☐	☐

Bitte kreuze den Strich auf der Linie an, der zeigt, wie viel Angst Du in dieser Situation hast:

keine Angst ┼──┼──┼──┼──┼──┼──┼──┼──┼──┼ sehr viel Angst

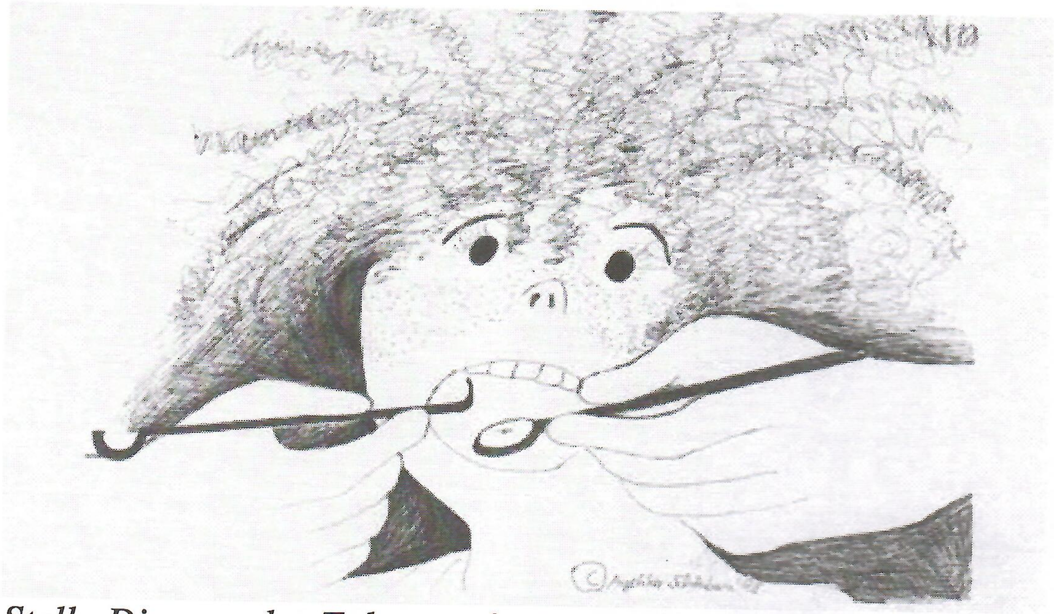

Stelle Dir vor, der Zahnarzt fängt mit der Untersuchung an. Du liegst im Behandlungsstuhl, Dein Mund ist weit offen. Der Zahnarzt beugt sich über Dich und steckt Instrumente in Deinen Mund.
*Kreuze an, was Du **jetzt** denkst und fühlst.*

	stimmt	stimmt nicht
21. Ich kann das Geräusch des Zahnbohrers nicht ertragen.	☐	☐
22. Ich muss daran denken, was alles noch geschehen könnte.	☐	☐
23. Ich mache die Augen ganz fest zu.	☐	☐
24. Ich habe ganz starkes Herzklopfen.	☐	☐
25. Ich halte mich am Behandlungsstuhl ganz fest.	☐	☐

Bitte kreuze den Strich auf der Linie an, der zeigt, wie viel Angst Du in dieser Situation hast:

keine Angst ├──┼──┼──┼──┼──┼──┼──┼──┼──┤ sehr viel Angst

☺

Vielen Dank für Deine Mitarbeit!
